# Genome Sequences of *Gordonia terrae* Phages Attis and SoilAssassin

**DOI:** 10.1128/genomeA.00591-16

**Published:** 2016-06-30

**Authors:** Welkin H. Pope, Daniel N. Biery, Zachary T. Huff, Amy B. Huynh, William M. McFadden, Julia S. Mouat, Scott E. Schneiderman, Hannah Song, Leah E. Szpak, Melanie S. Umbaugh, Brian A. German, Jill E. McDonnell, Nadia Mezghani, Claire E. Schafer, Paige K. Thompson, Megan C. Ulbrich, Victor J. Yu, Emily C. Furbee, Sarah R. Grubb, Marcie H. Warner, Matthew T. Montgomery, Rebecca A. Garlena, Daniel A. Russell, Deborah Jacobs-Sera, Graham F. Hatfull

**Affiliations:** Department of Biological Sciences, University of Pittsburgh, Pittsburgh, Pennsylvania, USA

## Abstract

Attis and SoilAssassin are two closely related bacteriophages isolated on *Gordonia terrae* 3612 from separate soil samples in Pittsburgh, PA. The Attis and SoilAssassin genomes are 47,881 bp and 47,880 bp, respectively, and have 74 predicted protein-coding genes, including toxin-antitoxin systems, but no tRNAs.

## GENOME ANNOUNCEMENT

*Gordonia* spp. are implicated in foaming of sludge in wastewater treatment plants and are identified as opportunistic pathogens in hospital infections (1–4). Seventeen bacteriophages of *Gordonia* have been isolated, sequenced, and deposited in GenBank (5–9). It is unclear if the phages’ genomic relationships reflect those of other phages of the phylum *Actinobacteria*, notably those of *Mycobacterium smegmatis* mc^2^155 whose phages exhibit a continuum of genetic diversity (10–16). The Science Education Alliance-Phage Hunters Advancing Genomics and Evolutionary Science (SEA-PHAGES) program is a course-based research experience in which undergraduates are immersed in research, using phage isolation and bioinformatics as a method to fuse authentic research and education (17). SEA-PHAGES has recently expanded its range of hosts for phage isolation to *Gordonia terrae* 3612.

Attis and SoilAssassin were isolated from separate soil samples at the University of Pittsburgh through direct plating of filtered soil extracts on *G. terrae* 3612. The phages were plaque purified and electron microscopy revealed that both phages have long non-contractile tails and isometric heads. DNA was isolated, and sequenced using Illumina MiSeq technology with 140 bp single-end reads. Reads were assembled using Newbler into major contigs for each phage of 47,881 bp and 47,880 bp with 949-fold and 932-fold coverages for Attis and SoilAssassin, respectively. Both have G+C% content of 66.8%, and discrete genome ends with 11-base single-stranded 3′ extensions (5′-TACCAGGGGGA). BLASTn alignment of the two genomes shows that they differ by only a single base substitution and single bp insertion in Attis. Protein-coding genes were predicted using Glimmer (18), GeneMark (19), DNA Master (http://cobamide2.bio.pitt.edu), and Phamerator (20), and functions were assigned to 35 of the 74 genes in each genome using BLAST (21) and HHPred (22, 23) alignment against the publically available databases GenBank, the Protein DataBase, and pFamA. Predicted functions include those for virion structure genes, a tyrosine integrase and immunity repressor, a RecET recombination system, RusA resolvase, and two HNH endonucleases.

Attis and SoilAssassin form plaques with distinct morphologies. SoilAssassin plaques are large (4-mm diameter), whereas Attis forms predominantly smaller plaques (1-mm diameter) although a few larger plaques are also observed. We predict that the single bp deletion at coordinate 25,254 of SoilAssassin in a putative minor tail protein gene is responsible for the plaque size difference. We note that Attis *29* likely corresponds to the parental form of the gene as the entire coding sequence is related to genes in several other distant phages. This relationship is reminiscent of the side tail fiber gene frameshift mutation in PaPa strains of phage lambda that facilitate large plaque formation relative to its Ur-lambda parent, which adsorbs to cells more rapidly than lambda PaPa thus reducing plaque size (24). Attis and SoilAssassin *29* genes are located at the 3′ end of the virion structure and assembly operons, consistent with encoding tail fiber proteins.

Both phages encode putative tyrosine integrase and repressor genes near the center of their genomes, and are predicted to integrate into a tRNA^ser^ gene corresponding to GTR9_RS02590 in *Gordonia* sp. KTR9.

### Nucleotide sequence accession numbers.

The Attis and SoilAssassin genomes are available from GenBank under accession numbers KU963247 and KU963246, respectively.
